# Gene-guided Gefitinib switch maintenance therapy for patients with advanced EGFR mutation-positive Non-small cell lung cancer: an economic analysis

**DOI:** 10.1186/1471-2407-13-39

**Published:** 2013-01-29

**Authors:** Jun Zhu, Te Li, Xiaohui Wang, Ming Ye, Jian Cai, Yuejuan Xu, Bin Wu

**Affiliations:** 1 Department of Pharmacy, Shanghai Chest Hospital, affiliated with the School of Medicine, Shanghai Jiaotong University, West Huaihai Road 241, Shanghai, China; 2 Department of Pharmacy, Yuxi People’s Hospital, affiliated with the Kunming Medical College, Nieer Road 21, Yuxi, China; 3 Department of Clinical Oncology, Renji Hospital, affiliated with the School of Medicine, Shanghai Jiaotong University, Dongfang Road 1630, Shanghai, China; 4 Department of Clinical Oncology, Taixing People’s Hospital, affiliated with the School of Medicine, Yangzhou University, Changzheng Road 1, Taixing, China; 5 Department of Clinical Oncology, the Second Hospital of Nanjing, affiliated with the Medical School of South East University, Zhongfu Road 1, Nanjing, China; 6 Medical Decision and Economic Group, Department of Pharmacy, Renji Hospital, affiliated with the School of Medicine, Shanghai Jiaotong University, Dongfang Road 1630, Shanghai, China

**Keywords:** Gefitinib maintenance treatment, EGFR mutation, Cost-effectiveness, Non-small cell lung cancer

## Abstract

**Background:**

Maintenance therapy with gefitinib notably improves survival in patients with advanced non-small cell lung cancer (NSCLC) and EGFR mutation-positive tumors, but the economic impact of this practice is unclear.

**Methods:**

A decision-analytic model was developed to simulate 21-day patient transitions in a 10-year time horizon. The clinical data were primarily obtained from the results of a pivotal phase III trial that assessed gefitinib maintenance treatment in patients with advanced NSCLC. The cost data were derived from the perspective of the Chinese health care system. The primary outcome was the incremental cost-effectiveness ratio (ICER) at a willingness-to-pay (WTP) threshold of 3 times the per capita GDP of China. Sensitivity analyses were used to explore the impact of uncertainty regarding the results. The impact of the gefitinib patient assistance program (GPAP) was evaluated.

**Results:**

After EGFR genotyping, gefitinib maintenance treatment for advanced NSCLC with EGFR mutations increased the life expectancy by 0.74 years and 0.46 QALYs compared with routine follow-up at an additional cost of $26,149.90 USD ($7,178.20 with the GPAP). The ICER for gefitinib maintenance was $57,066.40 and $15,664.80 per QALY gained (at a 3% discount rate) without and with the GPAP, respectively. The utility of progression free survival, the hazard ratio of progression-free survival for gefitinib treatment and the cost of gefitinib per dose were the three factors that had the greatest influence on the results.

**Conclusions:**

These results indicate that gene-guided maintenance therapy with gefitinib with the GPAP might be a cost-effective treatment option.

## Background

Lung cancer is the most prevalent malignant cancer in adults, with over 1.3 million deaths from the disease per year [1]. Non-small cell lung cancer (NSCLC) accounts for nearly 85% of all cases of lung cancer [2]. Locally advanced or metastatic NSCLC accounts for approximately 46% of cases at the time of presentation [3]. The current treatment guidelines recommend four to six cycles of first-line platinum-based doublet chemotherapy for advanced NSCLC [4]. However, the median overall survival (OS) time is still approximately 10 months, and even in the most favorable situations, most patients die within two years. Clearly, the poor clinical outcomes of advanced NSCLC present a challenge for oncologists to improve the clinical benefits of new treatment for patients before disease progression.

The role of maintenance therapy in patients who remained progression free after first-line chemotherapy has been well established by several Phase III trials [5-11]. At present, pemetrexed and erlotinib have been approved for the maintenance treatment of advanced NSCLC in Europe and the USA [12]. Although gefitinib, an EGFR tyrosine kinase inhibitor (TKI), failed to show a significant survival benefit with the addition of gefitinib to platinum-based chemotherapy [13,14]. However, it has been recommended as a first-line regimen for treating advanced NSCLC with EGFR mutations due to its more favorable health outcomes compared with platinum-based chemotherapy [15,16]. In patients with pretreated advanced NSCLC, gefitinib showed the noninferiority in comparison with docetaxel for overall survival [17]. One recent phase III clinical study examined gefitinib as a maintenance therapy in patients who attained tumor control with first-line chemotherapy [11]. Li Z. and colleagues found that progression-free survival (PFS) was significantly longer with gefitinib than with placebo. In patients with tumors bearing an EGFR mutation, the median PFS reached 16.6 months (HR 0.17, 95% CI 0.07–0.42). By contrast, PFS was not significantly different between the gefitinib and control arms for patients with EGFR mutation-negative tumors (HR 0.86, 0.48–1.51). This comes at a cost of substantially higher drug expenses due to the high price of gefitinib. Limiting this treatment to patients with EGFR mutation-positive tumors might be one potential way to improve the economic outcome of gefitinib maintenance treatment.

Health resource allocation decisions are based increasingly on economic analyses that identify the therapies that provide the greatest health benefits at acceptable costs, especially in a health resource-limited setting. Because clinical trials rarely include economic health assessments, mathematical modeling is widely used to perform economic health analyses, particularly for extrapolating to timepoints beyond trial durations. The objective of our study was to compare the economic outcome of gene-guided gefitinib maintenance treatment with the routine follow-up following first-line platinum-based chemotherapy for advanced NSCLC with EGFR mutation from the perspective of the Chinese health care system. Although erlotinib and pemetrexed have been used for maintenance treatment, the current analysis would not include other treatment arms due to no clinical trials for directly comparing the clinical outcomes of gefitinib, erlotinib and pemetrexed.

## Methods

### Analytical overview and model structure

**Figure 1:**
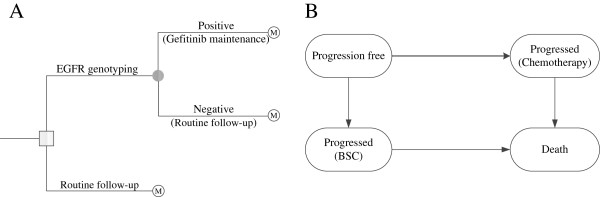
The schematics of the decision tree (A) and the Markov state transition model (B).

An economic model was constructed to analyze the ten-year clinical and economic outcomes of gefitinib maintenance therapies for patients with advanced NSCLC. Patients were assumed to either initiate observation with routine follow-up or to initiate maintenance treatment with gefitinib if the EGFR mutation screening was positive (Figure 1A). Health outcomes and costs were modeled using a Markov cohort model (Figure 1B) with four health states: progression free survival, progressed survival with supportive care, progressed survival with 2nd-line chemotherapy and death. In Markov models, a patient is always in one of a series of distinguished health states, called Markov states. All events are represented as movements from one state to another [18,19]. The cycle length of the model was 3 weeks. The risk of PFS and OS for patients in the model was determined according to the FS and OS survival data reported in clinical trials [11,20]. The R statistical environment (version 2.15.0; R Development Core Team, Vienna, Austria) was used to develop and solve the model. This economic study was based on a literature review and model techniques, and did not require approval by the institutional Research Ethics Board.

We assumed that the clinical characteristics of the hypothetical cohort were similar to those reported by Zhang L. *et al*. [11] All patients with histologically or cytologically confirmed stage IIIb or IV NSCLC had completed four cycles of first-line platinum-based doublet chemotherapy, and they exhibited no disease progression or unacceptable toxic effects. They were 18 years or older and the WHO performance status was 0–2. Except the four cycles of first-line platinum-based doublet chemotherapy, no other therapeutic agent was previously administered. The initial health status of the patients was progression free. Two competing strategies for these patients were compared: 1) routine follow-up for all patients (Control strategy) and 2) routine follow-up plus gefitinib maintenance for patients with EGFR mutation-positive tumors and routine follow-up only for patients with EGFR mutation-negative tumors (Gefitinib strategy). After the cancer progressed, patients were treated with 2nd-line chemotherapy or supportive care. To simplify the model, we assumed there is no possible to go from “progressed” back to “disease free” upon treatment [21,22].

The analysis was conducted from the perspective of the Chinese healthcare system. The costs are presented in 2012 US dollars. The outcomes calculated for each strategy included progression free life years, overall life years (LYs), quality-adjusted life years (QALYs) and the costs of advanced NSCLC care. The results are reported as the incremental cost-effective ratio (ICER) over the 10-year period calculated using the model. The costs and QALYs were each discounted at an annual rate of 3%.

### Clinical data and adjusted indirect comparisons

The transition parameters and proportions were based on a meta-analysis or randomized clinical trials to the greatest possible extent.

Kaplan-Meier survival curves for PFS for each strategy were taken from the pivotal gefitinib maintenance clinical trials [11]. A total of 296 patients with advanced NSCLC without disease progression after first-line chemotherapy were enrolled in this trial and randomly assigned 1:1 to receive either the Gefitinib strategy or the Control strategy. In this report, nearly 50% of patients tested were deemed EGFR mutation-positive; 27% of the study population had tumor samples available for EGFR mutation analysis. The survival analysis demonstrated that the median PFS for patients with EGFR mutation-positive tumors was significantly longer in the gefitinib maintenance arm than in the control arm (16.6 vs. 2.8 months, respectively, p < 0.001). The hazard ratio (HR) of PFS for gefitinib maintenance against the control arm for patients with EGFR mutation-positive tumors was 0.17 (95% CI: 0.07–0.42), but the EGFR mutation-negative subgroups did not differ significantly. The incidence of adverse events was similar between the two arms (p > 0.05). The cumulative probabilities of serious adverse events (SAEs, grade 3–4) in the gefitinib maintenance and control arms were 7% and 3%, respectively.

After disease progressed, patients would receive either 2nd-line chemotherapy or supportive care. The proportion of patients receiving 2nd-line chemotherapy was derived from literatures [5-10]. Kaplan-Meier overall survival (OS) curves for 2nd-line chemotherapy and supportive care were obtained from the trial reported by Shepherd FA and colleagues [20]. The median OS periods were 7.5 and 4.6 months in the 2nd-line chemotherapy and control arms, respectively. Weibull curves were fitted to the data extracted from the Kaplan-Meier curves using R statistical software because the Weibull distribution provided better fits to survival data than did other models [23-25]. The estimated scale and shape parameters, standard errors (SEs), adjusted R^2^ and correlation coefficients are presented in Table 1. The shape parameter (γ) allows the hazard function to increase or decrease with increasing time; if γ > 1.0, the hazard rate strictly increases in a nonlinear pattern with increasing time. The scale parameter (λ) is related to the unit of time measurement. The survival probability at time *t* could be calculated by following formula: *S*(*t*) = P(*T* ≥ *t*) = exp(−*λt*^*γ*^). The transition probability at current cycel *t* could be calculated by following formula:

(1) $$P\left(t\right)=1-\exp\left[\lambda {\left(t-1\right)}^{\gamma }-\lambda t^{\gamma }\right]$$

**Table 1:** Clinical data

**Parameter**	**Values**	**Description and references**
Weibull survival model of PFS in the Control strategy	Scale = 0.1559;	[11]
	Shape = 1.045;	
	r^2^ = 0.976	
Weibull survival model of OS for supportive care	Scale = 0.04006;	
	Shape = 1.156;	[20]
	r^2^ = 0.9898	
Weibull survival model of OS for 2nd-line chemotherapy	Scale = 0.03897;	[20]
	Shape = 1.509;	
	r^2^ = 0.981	
HR of PFS for the Gefitinib strategy in patients with an EGFR mutation	0.17 (95% CI:0.07–0.42)	[11]
Frequency of EGFR mutations	50% (range: 8%–70%)^*^	[11]
Proportion of patients receiving 2nd-line chemotherapy	56.6% (range: 26%–72%)^*^	[5-10]
Frequency of follow-up		
0–2 years	Once per four months	[26]
after 2 years	Once per year	[26]
Probability of SAEs in the Gefitinib strategy	7% (range: 5.25%–8.75%)^*^	[11]
Probability of SAEs in the Control strategy	3% (range: 2.25%–3.75%)^*^	[11]
Probability of SAEs using platinum-based chemotherapy	80% (range: 60%–100%)^*^	[27]

* The range was assumed for one-way sensitivity analysis.

### Cost and utility

The costs were estimated from the perspective of the Chinese health care system. Indirect costs were not included in this analysis. The direct medical costs considered in the model were: the detection of EGFR mutation, maintenance and 2nd-line chemotherapy (including prescription, preparation, and administration), concomitant medication during therapy, managing treatment-related SAEs, routine follow-up and laboratory tests.

The cost of EGFR genotyping per patient was provided by the AstraZeneca Innovation Centre China, Shanghai laboratory. The estimated treatment costs were based on the following schedules: gefitinib (250 mg per day) would be administered to patients with progression free survival after initial chemotherapy until the disease progressed. After the cancer progressed, 2nd-line chemotherapy and supportive care would be available. Based on the reported clinical trials, nearly 56.6% (26%-72%) of patients would receive 2nd-line chemotherapy regardless of the first-line treatment [5-10]. Of those, 50% of patients were given docetaxel ($1,942.4 per cycle), 20% gefitinib ($1,921.1 per cycle),15% erlotinib ($2,265.5 per cycle), and 15% were given pemetrexed ($4,383.3 per cycle) according to the expert opinions of Chinese oncologists. Patients would receive four median cycles of 2nd-line chemotherapy. The costs of four 2nd-line chemotherapies were derived from a previously published study, which estimated the cost of each 2nd-line drug treatment regimen for Chinese patients with NSCLC [28]. The utilization of resources related to supportive care, such as pain/sedation intervention, cachexia intervention, palliative radiotherapy and traditional Chinese medicine, was calculated from the records of 109 patients who received supportive care. In addition, the current analysis also included the cost of palliative care in end-of-life treatment, which was estimated from the records of 91 patients who died from NSCLC. Our analyses included the SAE treatment costs. The cumulative probabilities of SAEs were obtained from clinical trials, and we assumed that these events occurred with the same probabilities in every cycle. Due to the absence of cost data associated with adverse events in maintenance therapy, the costs of SAEs were calculated as the cumulative probabilities of the weighted average of first-line standard strategy SAE costs by the following formula: cost of SAEs in platinum-based chemotherapy per cycle × cumulative probability of SAEs in maintenance strategy / cumulative probability of SAEs in platinum-based chemotherapy.

Because it can be a challenge for patients to afford gefitinib in China, the Gefitinib Patient Assistance Program (GPAP) supplied by the pharmaceutical producer was introduced to make gefitinib available to eligible patients. Currently, the GPAP requires NSCLC patients to pay for six months of gefitinib, after which they receive donations of gefitinib until the end of their treatment. Therefore, the scenario analyses evaluated the importance of GPAP for gefitinib.

The utility values of the progression free survival and survival with disease progression were derived from previously published studies, and 0.65 and 0.47 were assigned, respectively. The standard errors were estimated at 25% of the mean in our sensitivity analysis [29].

Expected Cost and effectiveness(QALY and LY) accrued for the entire Markov process is the total number of cycles spent in each health state, each multiplied by the cost and effectiveness for that state.

### Sensitivity analyses

The median PFS and OS time of advanced NSCLC would not exceed one and two years, and most of patients would die within five years [30]. In base case analysis, the timeframe of 1 (scenario 1), 2 (scenario 2) and 5 (scenario 3) years was used to test the impact of observational period on the model outputs. The influences of each parameter value in the model were examined through one-way sensitivity analyses. The results of these analyses are presented as a tornado diagram depicting the lower and upper values for the cost-effectiveness ratios of the Gefitinib strategy versus the Control strategy for each varied model input, which are listed and illustrated in Table 1 and Table 2. A probabilistic sensitivity analysis (PSA) was performed to examine the uncertainty related to which strategy has the greatest likelihood of being cost-effective by randomly sampling the parameters from defined distributions. The model used log-normal distributions for costs and beta distributions for utility values and probabilities or proportions with an assumed standard deviation of 25% from the mean values when reported data were not available. Using these distributions, one thousand iterations of the model were conducted to generate the total cost and QALY distributions for each strategy. The net monetary health benefit (NMHB) was used to indicate the economic outcome of each strategy for any iteration. The NMHB varies depending on the value of willingness to pay (WTP). The current analysis used three times the per capita GDPs of China and Shanghai City as the thresholds according to the World Health Organization (WHO) guidelines for cost-effectiveness analysis [31-33]. The probability of an NMHB for each strategy can be measured by comparing the number of achieving the greatest NMHB across all 1000 iterations. The results are shown as a cost-effectiveness acceptability curve (CEAC) with varied WTP values in a range from US $0/QALY to US $100,000/QALY.

**Table 2:** Base-case costs estimates ($, year 2012 values) and utilities

**Parameter**	**Median**	**Range**	**Description and references**
Cost of EGFR genotyping per patient	507.9	381–634.9	Local charge
Cost of gefitinib per 250 mg ($)	77.8	38.9–77.8^*^	Local charge
Cost of follow-up per unit ($)	55.6	41.7–69.4	[34]
Cost of 2nd-line chemotherapy per cycle ($)	2352.7	1921.1–4383.3	Calculation
Cost of palliative care in end-of-life treatment ($)	3664.3	21.4–48750.2	Calculation
Cost of supportive care per cycle ($)	337.5	158.7–793.7	Calculation
Cost of SAEs in platinum-based chemotherapy per cycle ($)	507.4	189.7–825.0	Calculation
Expenditures of SAEs in maintenance treatment per cycle			
Cost of SAEs in Gefitinib strategy per cycle ($)	Formula^#^		Calculation
Utilities			
Utility of PFS	0.65	0.26–0.87	[29]
Utility of OS	0.47	0.19–0.58	[29]

* The range was assumed for a one-way sensitivity analysis.

# Formula: Cost of SAEs in platinum-based chemotherapy per cycle × Cumulative probability of SAEs in maintenance strategy / Cumulative probability of SAEs in platinum-based chemotherapy.

## Results

### Base-case analysis

The model results indicate that imitating gefitinib maintenance treatment increased the health benefits for patients who had completed standard first-line chemotherapy. Increased progression free LYs appeared after 1, 2, 5 and 10 years (gained an additional 0.22, 0.45, 0.69 and 0.74 years, respectively), and LYs increased by 0.16, 0.41, 0.69 and 0.74 years, respectively. The additional QALYs gained ranged from 0.12 at 1 year to 0.46 at 10 years (Table 3). The increased costs of the Gefitinib strategy without or with the GPAP were $10,794.00 and $5,999.10 at 1 year to $26,149.90 and $7,178.20 at 10 years, respectively. The ICER for gefitinib maintenance was $57,066.40 per QALY gained and $35,260.10 per LY gained at 10 years. When the GPAP was included, the ICER decreased to $15,664.80 per QALY gained and $9,678.90 per LY gained.

**Table 3:** Summary of the cost and outcome results in base-case analysis

**Strategy**	**Cost($)**	**Progression free LYs**	**Overall LYs**	**QALYs**	Incremental cost per QALY*
1 year (scenario 1)					
Control	2,981.3	0.35	0.51	0.30	
Gefitinib without GPAP	13,775.3	0.56	0.67	0.42	92,968.5
Gefitinib with GPAP	8,980.4	0.56	0.67	0.42	51,669.9
2 year (scenario 2)					
Control	4,545.6	0.36	0.57	0.33	
Gefitinib without GPAP	22,063.0	0.81	0.97	0.60	65,514.8
Gefitinib with GPAP	10,660.6	0.81	0.97	0.60	22,870.0
5 year (scenario3)					
Control	4,913.2	0.36	0.57	0.33	
Gefitinib without GPAP	29,705.8	1.06	1.26	0.76	57,788.9
Gefitinib with GPAP	11,884.7	1.06	1.26	0.76	16,249.9
10 year					
Control	4,917.0	0.36	0.57	0.33	
Gefitinib without GPAP	31,066.9	1.11	1.31	0.79	57,066.4
Gefitinib with GPAP	12,095.2	1.11	1.31	0.79	15,664.8

* Compared with Control strategy.

### Uncertainty analyses

**Figure 2:**
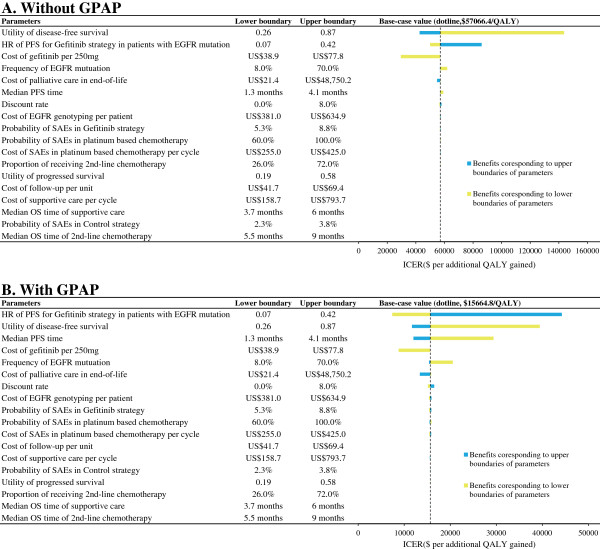
One-way sensitivity analyses show the lower and upper values for the cost-effectiveness ratio of the Gefitinib strategy to the Control strategy for each parameter.

The one-way sensitivity analyses showed that some model variables had a substantial impact on the results; these are presented in the tornado graphs in Figure 2. Regardless of the GPAP, the two most influential variables were the utility of progression free survival and the HR of PFS for the Gefitinib strategy in patients with an EGFR mutation. The cost of gefitinib per dose, the frequency of EGFR mutations and the cost of palliative care in end-of-life treatment had a medium impact on the ICER. Other parameters, such as the cost, median OS time of 2nd-line chemotherapy or supportive care, and probability of SAEs, had little sensitivity on the model outputs. With GPAP, model output was moderately sensitive to the median PFS time of control strategy.

**Figure 3:**
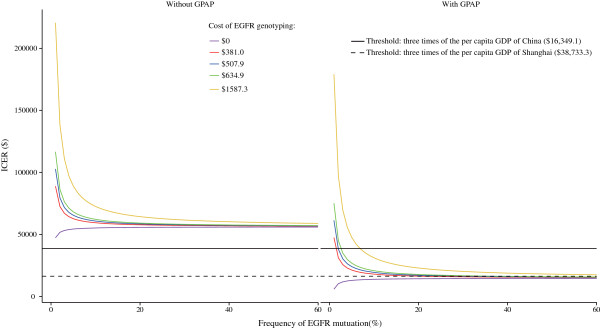
Two-way sensitivity analysis of the effects of the frequency of EGFR mutations and the cost of EGFR genotyping.

A two-way sensitivity analysis incorporating the frequency of EGFR mutations and the cost of EGFR genotyping was performed. This analysis indicated that gefitinib maintenance was more cost-effective in the population with a higher rate of EGFR mutation-positive advanced NSCLC. The ICER was sensitive to rates from approximately 7% to 20%. A lower cost of detecting EGFR mutations would improve the ICER values of the Gefitinib strategy. However, the impact was small (Figure 3).

**Figure 4:**
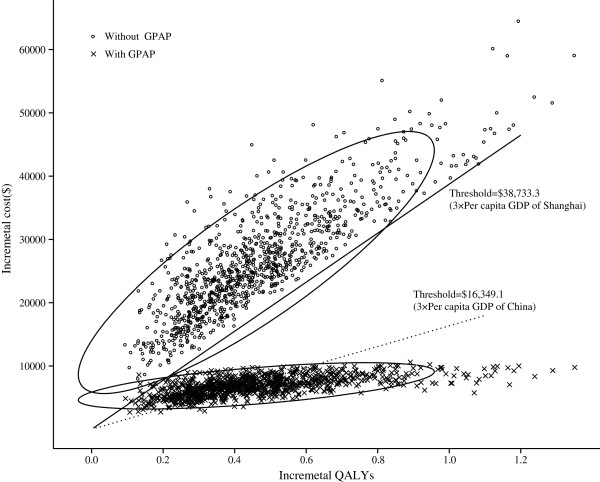
**A probabilistic scatter plot of the incremental cost-effectiveness ratio (ICER) between the Control and Gefitinib strategies for a cohort of 1,000 patients.** Each dot represents the ICER for 1 simulation. An ellipse surrounds 95% of the estimates. Dots that are located below the ICER threshold represent cost-effective simulations for the active strategy compared with the Control strategy.

**Figure 5:**
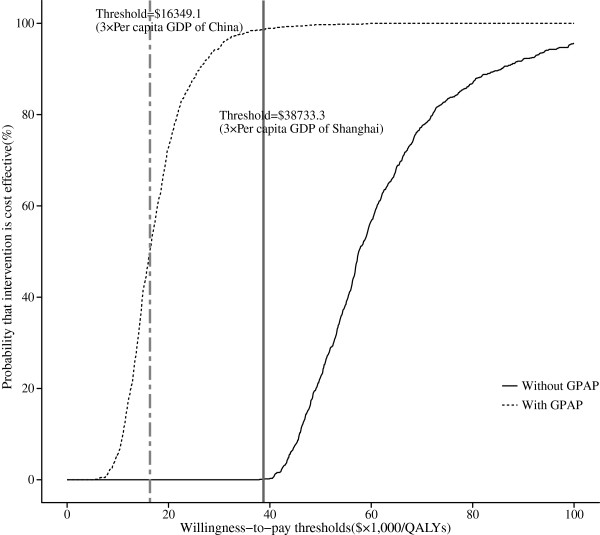
The cost-effectiveness acceptability curves showing the probabilities of net benefits achieved by the Gefitinib strategy compared to the Control strategy at different WTP thresholds in advanced NSCLC patients.

When no GPAP was supplied, the probabilistic sensitivity analysis showed a nearly zero cost-effective probability even at a threshold of $38,733.30 (Figure 4). In the GPAP setting, when the threshold was equal to three times the per capita GDPs of China ($16,349.10) and Shanghai ($38,733.30) in 2011, nearly 51% and 99% of the advanced NSCLC cohort achieved cost-effectiveness, respectively. Correspondingly, the acceptability curves showed that the probability of cost-effectiveness also increased with an increase in the willingness-to-pay threshold, which was sensitive to the thresholds from approximately $41,000 to $100,000 in the no-GPAP setting and from approximately $9,700 to $35,000 in the GPAP setting (Figure 5).

## Discussion

Reports of a clinical benefit from gefitinib maintenance therapy after first-line platinum-based chemotherapy in clinical trials caused great excitement among both oncologists and patients. However, the widespread and long-term use of gefitinib comes with a dramatically increased burden on health resources, which is a concern for health policy decision makers. The need for a precise economic assessment of gefitinib maintenance use in this clinical setting is becoming urgent.

This work is the first study to address the cost-effectiveness of gene-guided gefitinib maintenance treatment after standard chemotherapy for patients with advanced EGFR mutation-positive NSCLC. Genotyping for EGFR mutations with the subsequent gefitinib maintenance treatment of patients with confirmed mutations yielded an average ICER of $57,066.40 per additional QALY gained against control strategy. This ratio is largely attributable to the higher costs associated with the acquisition of gefitinib, whereas the costs of EGFR genotyping and the costs of managing progressed disease had little influence. Finding of scenario analyses in Table 3 indicated that gefitinib would be more cost-effective (ICER without GPAP, $92,968.5/QALY at 1 year to $57,066.4/QALY at 10 year ) with the longer timeframe because the health benefit related to progression free survival yielded by gefitinib could be more obviously displayed (incremental progression free LYs, 2.2 at 1 year to 0.7 at 10 year; incremental overall LYs, 0.16 at 1 year to 0.74 at 10 year; incremental QALYs, 0.12 at 1 year to 0.46 at 10 year ), especially after two years. At one year, more patients in gefitinib arm was still in the state of progression-free survival and more patients in control arm had moved into progressed survival, which resulted in the gap between the incremental progression free LYs and the incremental overall LYs in gefitinib strategy comparing with control strategy. Pemetrexed switch maintenance treatment has been widely recommended for patients with advanced NSCLC. A pharmacoeconomic analysis from a US payer and the Swiss Health Care System perspective showed that the pemetrexed switch maintenance treatment resulted in an incremental cost of $122,371 per additional life year gained and $138,500 per additional QALY gained in patients with nonsquamous cell histology [22,35]. In comparison with pemetrexed switch maintenance treatment, gene-guided gefitinib maintenance has a much more favorable ICER, which is considerably contributed by the more favorable PFS time of gefitinib than pemetrexed in patients with EGFR mutations (16.6 months vs. 4.4 months) [8,11]. These results suggest that gefitinib maintenance tailoring for patients with EGFR mutations could deliver health benefits at a lower cost than pemetrexed switch maintenance therapy. This finding comes in line with the two recent economic studies, which provided the favorable economic evidence to support the first-line therapy with gefitinib for patients with EGFR mutation-positive and traditional chemotherapy for those with EGFR mutation-negative after mutation testing [36,37].

Although gefitinib maintenance yielded greater health benefits, the ICER did not approach the willingness-to-pay thresholds of $16,349.10 and $38,733.30 (3× the per capita GDPs of China and Shanghai in 2011, respectively). If the Gefitinib Patient Assistance Program were available to Chinese patients, the Gefitinib strategy might be a cost-effective alternative because the probability of cost-effectiveness reached nearly 51% at a threshold of $16,349.10 (Figures 4 and 5). For local governments in China, the per capita GDP differs significantly among the 32 provinces. In regions with a higher economic development level (3× the per capita GDP > $16,349.10), local health decision makers could consider covering gefitinib in their local supplemental medical service.

The ability of gefitinib to prevent disease progression in patients with EGFR mutation-positive tumors was a major determinant of clinical and economic outcomes. A one-way sensitivity analysis found that the most two sensitive parameters were the HR of PFS for the Gefitinib strategy in patients with EGFR mutations and the utility of progression free survival regardless of the use of the GPAP. This finding suggests that improving the quality of life, i.e., achieving the progression free state, could increase the cost-effectiveness of gefitinib maintenance treatment. At the same time, in patients who have a low risk of disease progression, such as adenocarcinoma histology, gefitinib maintenance treatment might be more cost-effective. The cost of gefitinib was another influential factor. When the price of gefitinib per 250 mg decreased by 50%, the ICERs for the Gefitinib strategy decreased to $29,493.40 and $8,792.60 per additional QALY gained without or with the GPAP, respectively. Although the PSA results indicated that the GPAP leads to the cost-effective probability of the Gefitinib strategy, approaching 51% at 3× the per capita GDP of China, a reduction in the price of gefitinib or a more preferential patient assistance program (i.e. pay for shorter than six months of gefitinib, after which they receive donations of gefitinib until the end of their treatment) might be the best strategies to achieve a more favorable ICER.

It is important to note that the current analysis did not evaluate the cost-effectiveness of gefitinib maintenance treatment for the whole cohort without EGFR genotyping. If all patients received gefitinib maintenance, the health outcome of 50% of the patients would not have improved because no statistically significant difference was found between the Gefitinib and Control strategies in patients who were EGFR mutation negative [11]. The cost of gefitinib for the whole cohort in the first 21-day cycle was nearly $1663.10, including $831.50 expended by patients who were EGFR mutation negative, which was higher than the cost of EGFR genotyping ($507.90) for the whole cohort. Thus, it was obvious that gefitinib maintenance without EGFR genotyping was not cost-effective when compared with gefitinib maintenance with EGFR genotyping and would become less cost-effective with a lower frequency of EGFR mutations due to the increased cost of gefitinib for patients who are EGFR mutation negative. We noticed that the frequency of EGFR mutations ranged from 8% in Caucasian patients to 30% in Asian patients. As a result, we concluded that gene-guided gefitinib maintenance treatment is superior to non-gene-guided treatment [38-40].

Other limitations of the study should also be considered. First, the present model did not include other EGFR-targeted agents used as maintenance treatments, such as erlotinib, to assess the incremental cost-effectiveness in comparison with gefitinib because no head-to-head trial data are currently available. Second, we did not conduct a budget impact analysis for the addition of gefitinib maintenance treatment on society. The annual incidence of lung cancer in China is approximately 300,000 cases [41]. Because the PFS of advanced NSCLC was nearly 58.9% after four cycles of standard chemotherapy and the frequency of EGFR mutations was 50%, gefitinib might be prescribed as a maintenance treatment to more than 88,000 patients each year [27]. Based on our model results, gefitinib maintenance treatment would result in a gain of approximately 24,700 QALYs and would increase expenditures by approximately $1,399 and $395 million without and with GPAP, respectively. Third, the current analysis incorporated PSF and OS data after cancer progression from different trials. Although the sensitivity of the OS data after cancer progression was little (Figure 2), the analysis should be updated when the overall survival of gefitinib maintenance therapy was available. Forth, some model inputs were obtained from literature published abroad due to a lack of Chinese data, such as the utility values. Fifth, the sensitivity and specificity of different genotyping facilities was not accounted. A new economic analysis of different genotyping facilities testing for EGFR mutations is necessary in the future. Finally, to simplify our evaluation, we did not include other adjuvant therapies, such as the traditional Chinese herbals for lung cancer. However, because the results of this analysis reflected the common clinical conditions of advanced NSCLC in China, we believe that this analysis can serve as an important reference for health policy decision makers.

## Conclusions

In the Chinese setting, gene-guided gefitinib maintenance treatment for patients with advanced NSCLC and EGFR mutation-positive tumors after first-line chemotherapy is indicated as a cost-effective chemotherapy option compared to routine follow-up based on its superior PFS benefit and the use of the Patient Assistance Program.

## Competing interests

The authors declare that they have no competing interests.

## Authors’ contributions

Jun Zhu and Te Li developed the economic model, performed the analyses and drafted the manuscript. Bin Wu contributed to the conception, design of the primarily model and interpreted the results. Xiaohui Wang and Ming Ye collected and reviewed data. Jian Cai provided clinical input, validated the model assumptions. Yuejuan Xu aided in defining economic setting. All authors read and approved the final manuscript.

## Pre-publication history

The pre-publication history for this paper can be accessed here:

http://www.biomedcentral.com/1471-2407/13/39/prepub
